# Challenges in Treating Schizophrenia with LAIs – Analysis of Own Results

**DOI:** 10.1192/j.eurpsy.2023.2209

**Published:** 2023-07-19

**Authors:** A. A. Todorov, M. Y. Stoimenova, T. R. Tzoneva, V. S. Tzankova

**Affiliations:** Psychiatry and medical psychology, Medical University - Pleven, Pleven, Bulgaria

## Abstract

**Introduction:**

Schizophrenia is a multifactorial and multifarious disorder with an unidentified etiology and pathogenesis, various clinical symptoms, unpredictable course and definite social significance. It’s social significance manifests in the long inability to work, the decrement of qualification and the burdening of family and social services. Patients find following therapy difficult; they often stop it altogether, which leads to new exacerbations and maintaining a vicious cycle.

**Objectives:**

To present an analysis of the results of the treatment of patients with LAIs – Trevicta and Xeplion in General Psychiatry Ward of UMBAL "Dr. Georgi Stranski” - Pleven for the period October 2016 – October 2022.

**Methods:**

Our retrospective research includes 17 patients treated in General Psychiatry Ward of UMBAL “Dr. Georgi Stranski”- Pleven. A treatment with LAI’s – Trevicta and Xeplion (Paliperidone palmitate) had been initiated in these patients. In consideration of the correct applications of the medication, they are always made by qualified personnel who received training for that purpose in the Ward. For assessment of the patients’ condition are used: Positive and Negative Syndrome Scale (PANSS) and Personal Social Performance (PSP). Standard statistic methods are used for processing results.

**Results:**

Our research includes 17 female patients as the Ward’s profile is such, with the average age of 38.65 years at the initiation of the treatment with Xeplion. Some were later introduced to Trevicta. Of all who participated in the research, 12 patients continue their treatment with regular applications, 2 have changed their address and by unreliable data still continue their treatment and for the other 3 we have no information of their status of treatment. The average count of admissions in the Ward before the initiation of treatment with LAI’s is 3.88 against 0.88 after the initiation. Regarding the PANSS scale – all score in the interval 75-100 points before initiation as opposed to the scores marked after the initiation of the medication that vary in the interval 34-48 points with definite distinctions in P and G items. Regarding the PSP scale – all participants score between 50 and 70 points before the initiation of the treatment as opposed to the scores of 89-95 points after the initiation. During the regular check-ups the patients report of subjective improvement, coupled with reports of objective and positive improvement by family.

**Conclusions:**

Treatment with LAIs – Xeplion and Trevicta has an unquestionable positive and lasting effect in the treatment of schizophrenia. Proof of that is the definite improvement of the symptoms, the increased social engagement and interpersonal communication. Everything mentioned is a valid reason to direct the treatment of schizophrenia in the direction of preservation of the personality of the patients.

**Disclosure of Interest:**

None Declared

